# The relationship of large city out-of-hospital cardiac arrests and the prevalence of COVID-19

**DOI:** 10.1016/j.eclinm.2021.100815

**Published:** 2021-04-07

**Authors:** Kevin E. McVaney, Paul E. Pepe, Lauren M. Maloney, E.Stein Bronsky, Remle P. Crowe, James J. Augustine, Sheaffer O. Gilliam, Glenn H. Asaeda, Marc Eckstein, Amal Mattu, Roberto Fumagalli, Tom P. Aufderheide, Michael T. Osterholm, Writing group on behalf of the Metropolitan EMS Medical Directors Global Alliance

**Affiliations:** a Department of Emergency Medicine, University of Colorado School of Medicine, Denver, CO, USA; b Denver Health and Hospital Authority, Denver, CO, USA; c Dallas County Emergency Medical Services and County Public Safety Agencies, Dallas, TX, USA; d Broward Sheriff's Office, Ft. Lauderdale, FL, USA; e Palm Beach County Fire Rescue, West Palm Beach, FL, USA; f Department of Management, Policy and Community Health, School of Public Health, University of Texas Health Sciences Center, Houston, TX, USA; g Metropolitan EMS Medical Directors Global Alliance, Dallas, TX, USA; h Department of Emergency Medicine, Stony Brook University Hospital, Stony Brook, NY, USA; i Colorado Springs Fire Department, Colorado Springs, CO, USA; j ESO, Austin, TX, USA; k Wright State University, Dayton, OH, USA; l Fire Department of New York, New York, NY, USA; m Los Angeles Fire Department, Los Angeles, CA, USA; n Keck School of Medicine of the University of Southern California, Los Angeles, CA, USA; o University of Maryland, Baltimore, MD, USA; p Niguarda Hospital, University of Milano-Bicocca, Milan, Italy; q Agenzia Regionale Emergenza Urgenza (AREU), Lombardy, Italy; r Resuscitation Research Center, Medical College of Wisconsin, Milwaukee, WI, USA; s University of Minnesota Center for Infectious Disease Research and Policy, Minneapolis, MN, USA

## Abstract

**Background:**

Though variable, many major metropolitan cities reported profound and unprecedented increases in out-of-hospital cardiac arrest (OHCA) in early 2020. This study examined the relative magnitude of those increases and their relationship to COVID-19 prevalence.

**Methods:**

EMS (9-1-1 system) medical directors for 50 of the largest U.S. cities agreed to provide the aggregate, de-identified, pre-existing monthly tallies of OHCA among adults (age >18 years) occurring between January and June 2020 within their respective jurisdictions. Identical comparison data were also provided for corresponding time periods in 2018 and 2019.  Equivalent data were obtained from the largest cities in Italy, United Kingdom and France, as well as Perth, Australia and Auckland, New Zealand.

**Findings:**

Significant OHCA escalations generally paralleled local prevalence of COVID-19. During April, most U.S. cities (34/50) had >20% increases in OHCA versus 2018–2019 which reflected high local COVID-19 prevalence. Thirteen observed 1·5-fold increases in OHCA and three COVID-19 epicenters had >100% increases (2·5-fold in New York City). Conversely, cities with lesser COVID-19 impact observed unchanged (or even diminished) OHCA numbers. Altogether (*n* = 50), on average, OHCA cases/city rose 59% during April (*p* = 0·03). By June, however, after mitigating COVID-19 spread, cities with the highest OHCA escalations returned to (or approached) pre-COVID OHCA numbers while cities minimally affected by COVID-19 during April (and not experiencing OHCA increases), then had marked OHCA escalations when COVID-19 began to surge locally. European, Australian, and New Zealand cities mirrored the U.S. experience.

**Interpretation:**

Most metropolitan cities experienced profound escalations of OHCA generally paralleling local prevalence of COVID-19.  Most of these patients were pronounced dead without COVID-19 testing.

**Funding:**

No funding was involved. Cities provided de-identified aggregate data collected routinely for standard quality assurance functions.

**Research in context**

**Evidence before this study**

The highly stressful clinical challenge of out-of-hospital cardiac arrest (OHCA) occurs frequently and somewhat predictably with about 30,000 cases routinely presenting each month in North America and a similar proportionate number in European nations. However, as SARS-CoV2 infections began to surge in the first epicenters such as Milan, London, New York and Detroit, their emergency medical services (EMS) agencies were already reporting alarming increases in OHCA, even prior to implementation of shelter-at-home directives and the tallying of COVID-19 related deaths.

**Added value of this study**

This study further indicates that there was a direct association between the local prevalence COVID-19 and the frequency of OHCA across several of the largest European cities and 50 of the largest metropolitan areas in the U.S. that ordinarily account for over one-quarter the U.S. population. While profound escalations in OHCA were occurring across the majority these metropolitan cities, the findings also revealed that the proportionate magnitude of increases in OHCA, and any subsequent decreases, closely paralleled the concurrent local prevalence of COVID-19. In addition, despite local lockdown directives, cities not significantly impacted by COVID-19 at first did not experience any significant increases in OHCA until SARS-CoV2 eventually surged through their respective jurisdictions. In certain cities that were less impacted by COVID-19, the frequencies of OHCA were relatively diminished as predicted by contracted populations (fewer commuters, visitors, tourists). Of note, sudden sharp rises in OHCA cases typically signaled ensuing local surges in documented COVID-19 cases.

**Implications of all the available evidence**

Dramatic escalations in OHCA cases can immediately foreshadow and then parallel the local prevalence of COVID-19. OHCA is likely a direct consequence of SARS-CoV-2 infection and considering that testing and eventual documentation of COVID-19 may be somewhat delayed after disease onset, observations of sudden increased frequencies in OHCA may serve as a signal of COVID-19 surges and cluster areas. In most of the cases, these many thousands of additional OHCA cases were not resuscitated and not tested for COVID-19 and therefore not counted among COVID-19 related deaths.

Alt-text: Unlabelled box

## 1 Introduction

Sudden out-of-hospital cardiac arrest (OHCA) remains a leading cause of premature death in the United States (U.S.) and other developed countries, totaling nearly 1000 daily cases in North America alone [1,2]. While historically constituting only 1% of emergency medical services (EMS) responses (9–1–1 / 9–9–9 / 1–1–2 / 0–0–0 or equivalent), each OHCA creates a markedly disproportionate operational impact amplified by the exceptionally time-dependent and stressful nature of such clinically-challenging events [2], [3], [4].

Soon after shelter-at-home directives were instituted in mid- to late March 2020 in Europe and the U.S., EMS and emergency department (ED) teams experienced marked (30–40%) decreases in patient volumes, especially trauma-related responses [5,6]. However, EMS practitioners also recognized increased frequencies of OHCA responses [7], [8], [9]. Early epicenters such as Milan and New York City soon reported profound increases in OHCA that were straining their EMS resources severely [7,9]. By April 2020, increased OHCA numbers were being observed in other large cities beyond the initial epicenters. Concern existed that many sheltering persons developing acute coronary syndromes and reticent to seek help may have progressed to OHCA at home [10]. Although most clinicians initially considered COVID-19 primarily a pulmonary-focused disease, it was also being speculated that COVID-related hypercoagulable conditions might be precipitating lethal pulmonary thromboemboli (PTE) or even coronary occlusions [11], [12], [13], [14], [15].

For over two decades, jurisdictional EMS system lead physicians for 50 of the largest cities in the U.S. and their medical director counterparts in the United Kingdom (UK), European Union (EU), Australia, New Zealand, and other nations have formed a de facto networking alliance and study group that deliberates and exchanges best practices while also facilitating information-sharing for EMS professionals worldwide [16]. Beyond longstanding daily internet and mobile cross-messaging, these chief medical officers also initiated twice-weekly 90-minute teleconferences on February 20^th^, 2020, largely to address COVID-19 [16,17]. Within their respective jurisdictions, these physicians are medically responsible for approving EMS system protocols, related training, and hospital destination policies for ambulance and first responders crews, but also professional interface with the medical, public health and public safety sectors within their communities. They also steward EMS quality assurance and research efforts, not only for day-to-day emergency system responses, but also the medical aspects of homeland security and public health emergencies [18].

Considering combined residential (nighttime) populations of nearly 60 million and another net estimate of 25 to 40 million daily visitors, tourists and commuters to these metropolitan centers, this physician alliance oversees the front-line emergency care for what might ordinarily encompass one-quarter or more of the U.S. population [19,20]. Likewise, the UK, EU and other nations’ counterparts are collectively responsible for many additional tens of millions of residents, visitors, and commuters living, working or trafficking through their respective metropolitan cities [17], [18], [19], [20].

With many large cities beginning to observe operationally-stressful increases in OHCA by late March, the alliance's consensus was to formally study the issue. The initial primary aim was to quantify the relative increases in OHCA cases occurring in each of these largest U.S. cities during the first six months of 2020, and then compare those numbers to prior years (2018, 2019). Secondary aims were to compare findings with the estimated concurrent local prevalence of COVID-19, and then also compare U.S. findings to counterpart metropolitan cities globally.

## 2 Methods

### 2.1 Design

The EMS (9–1–1 system) medical directors for 50 of the largest U.S. cities (appendix) agreed to provide the aggregate, de-identified, pre-existing monthly tallies of adult (age ≥18 years) OHCA in their respective jurisdictions occurring between January and June 2020 supplemented by identical comparison data from corresponding time periods in 2018 and 2019. Integral to required public safety agency quality assurance programs, each participating city prospectively collects and reviews these OHCA data, most commonly using monthly reports. Corresponding data were requested from the largest city counterparts in Italy, UK and France, including Milan and London (well-publicized high COVID-19 attack rates) and Paris (intermediate attack rate) as well as Perth and Auckland (to examine non-U.S. regions with low COVID-19 attack rates).

Recognizing some slight variability in case inclusions across respective agencies, each jurisdiction was directed to provide data that were internally consistent from year to year. While most agencies routinely exclude or stratify the more identifiable and much less frequent precipitants of OHCA such as drowning, electrical shock, hangings, physical injury and toxicological causes, some agencies’ data may still contain one or more of these minor subcategories. In either situation, these identifiable etiologies only represent a very small percentage of OHCA cases and, for the central purpose of the year-to-year comparisons, the data included in this analysis from each site were internally consistent regardless of the inclusion criteria used by the respective jurisdictions. All authors have had, and continue to have, access to all of the de-identified aggregate data provided by each of the respective agencies.

Appreciating that locally-reported COVID-19 cases are affected by test availability, timing, technique, and reporting delays, the reported prevalence of COVID-19 was considered a best estimate of any actual concurrent prevalence. While numbers of hospitalizations or deaths may be a better gage, hospitalizations generally lag a week or more after symptom onset and deaths usually transpire 3 or 4 weeks later [21,22]. Accordingly, investigators garnered best-available concurrent case estimates using both U.S. Centers for Disease Control and Prevention (CDC) and New York Times COVID-19 databases. Cities were categorized for each respective month as experiencing very few COVID-19 cases (<5 per 100,000 population), intermediate levels of attack (5 to 50 per 100,000), or high levels of attack (>50 per 100,000) [21,22].

### 2.2 Setting

The combined 2020 best-estimate residential populations for catchment areas served by the contributing U.S. agencies totaled 56,084,562 with individual catchment populations ranging from 213,387 to 8,323,340 (mean, 1,121,691; median, 784,968). Accounting for pro-rated time-frames for commuters’ in-city presence and for the net numbers of at-home residential inhabitants, visitors, and tourists at any given time, these metropolitan EMS systems are, ordinarily, estimated to be responsible for about 80 to 95 million persons in these highly-trafficked cities [19,20]. Based on historical U.S. statistics (about one OHCA per 1000 population annually), this would ordinarily predict an estimated average of 7300 cases/month in these 50 cities.

### 2.3 Statistical analysis

For each metropolitan city, the historical OHCA monthly case counts were calculated using the corresponding averages of 2018 and 2019 monthly data. These monthly averages were used to determine the percent change in OHCA frequencies during each of the corresponding months in 2020 within each individual metropolitan city. Considering both existing monthly variations and the substantial operational impact of OHCA responses, the study group unanimously deemed, a priori, any percent changes >10% to be highly-significant differences. Absolute changes in OHCA numbers across all U.S. cities were also evaluated using paired t-tests in examining the monthly averages of cases per city.

### 2.4 Institutional review board

The Colorado Multiple Institutional Review Board (COMIRB) affiliated with the University of Colorado and Denver Health and Hospital Authority approved this study (COMIRB # 20–1339).

### 2.5 Role of funding sources

The source of data for this study was from public (government-based) agencies that provided de-identified aggregate data that are collected routinely as part of their respective quality assurance functions and therefore required no additional funding or extramural support.

## 3 Results

**Fig. 1:**
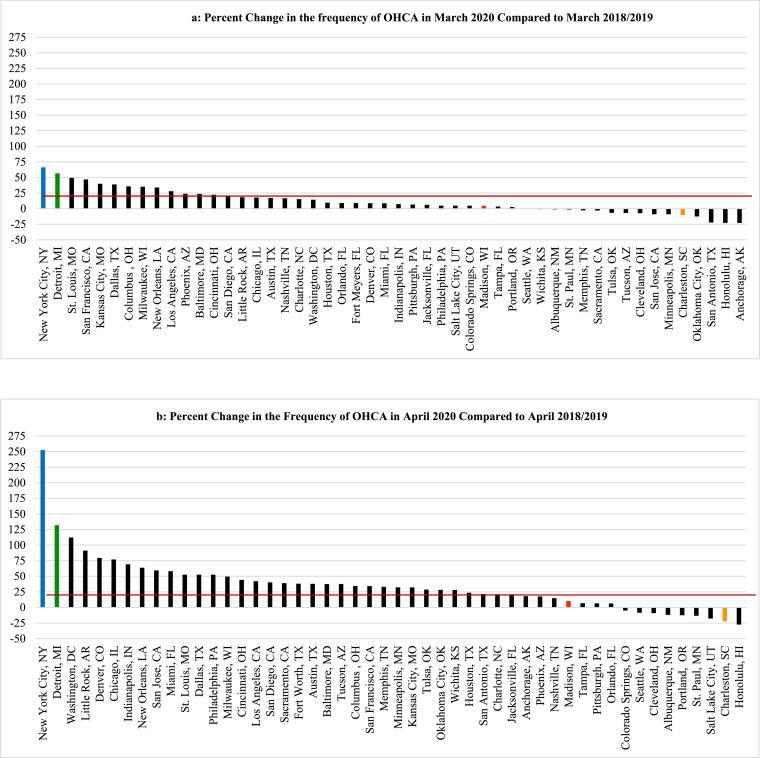

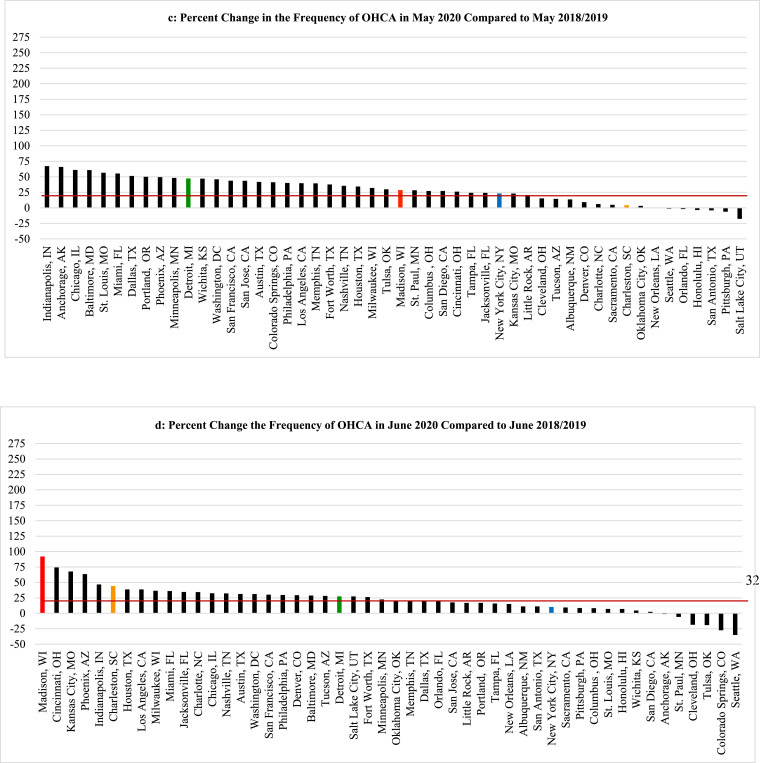
Comparing the total monthly numbers of out-of-hospital cardiac arrest (OHCA) for March, April, May and June 2020 to the average of the corresponding monthly totals for the prior two years (2018–2019), the percent change in monthly totals of OHCA cases from those prior years (y-axis) are displayed for each of 50 major U.S. cities (x-axis) during the months of March through June, respectively. Deviations of greater than 10% from prior years were deemed significant, both statistically and operationally. The red lines delineate cities with greater than 20% increases in OHCA over prior years.

**Fig. 2:**
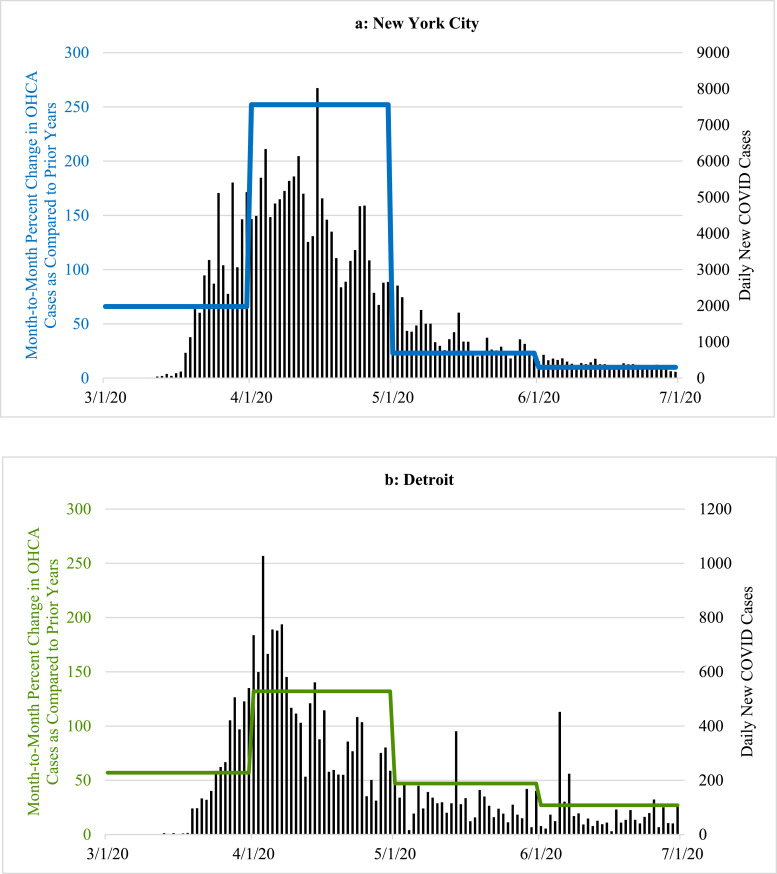

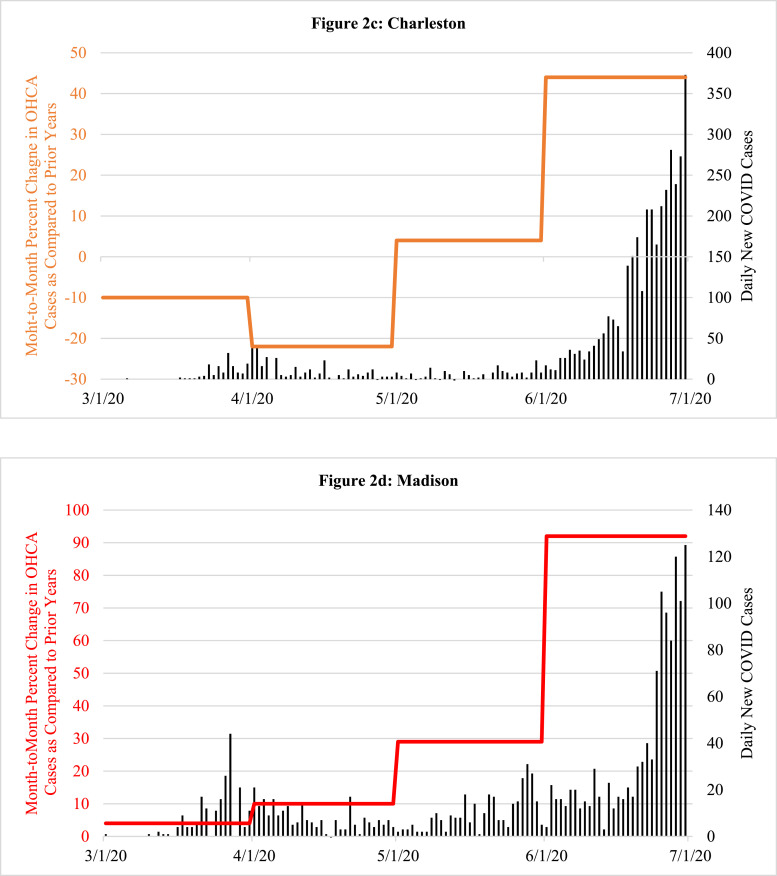
Examples of the percent change in the total monthly frequency of out-of-hospital cardiac arrest (OHCA) cases that were reported for March, April, May and June 2020, respectively, when compared to the averaged corresponding totals for previous two years. The colored horizontal lines, scaled on left y-axis, represent the overall averaged percent increase for each of the entire respective months. The overlay of the concurrent number of daily new COVID-19 cases (vertical black lines, scaled on right y-axis) helps to illustrate how the month by month increases or decreases in the numbers of OHCA cases in 2020 paralleled the prevalence of COVID-19 cases using four sample cities; two with a high prevalence of COVID-19 in March and April (New York City, Detroit) accompanied by a corresponding increase in OHCA that later diminished as the local prevalence of COVID-19 dissipated; and two other cities with late surges in OHCA that occurred along with corresponding increases in COVID-19 cases in late June 2020 (Charleston, Madison).

**Fig. 3:**
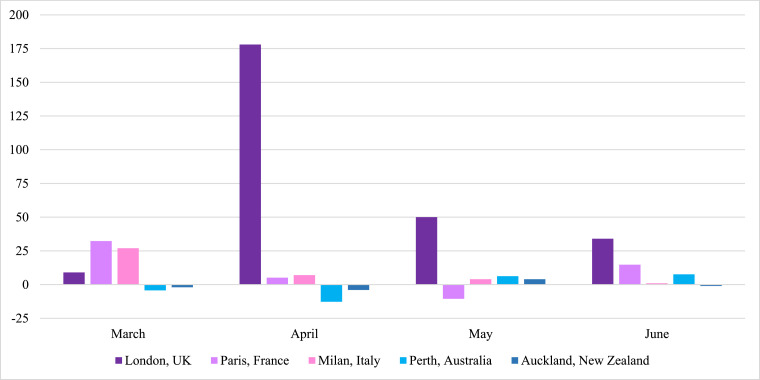
Month by month percent change in the total number of out-of-hospital cardiac arrest (OHCA) cases (y-axis) occurring in March, April, May and June 2020, respectively, when compared to the corresponding month by month average for the previous two years in several non-U.S. cities (x-axis).

The number of monthly cardiac arrests across the 50 U.S. cities remained relatively stable when comparing January 2020 with January 2018 and 2019, but a few cities began to observe upward trends in their leap year-adjusted numbers for February. By March 2020, significant increases in OHCA became evident in certain cities soon to be inundated by COVID-19 such as Milan, London, Paris, New York City and Detroit (Figs. 1a, 1b, 2a, 2b, 3).

Despite widespread stay-at-home directives and significantly fewer visitors, commuters and tourists, by April (Fig. 1b), the absolute number of OHCA cases across all 50 cities increased from 7257 (2018–2019 averages for April) to 11,564, a one-month increase of 4307 (59·3%) collectively. Altogether, the overall mean number of OHCA cases/city in April for all 50 cities combined rose from 144 mean cases per city (averaging 2018–2019) to 231 mean cases per city in 2020 (*p* = 0·03). Also during April, 34 of the 50 cities experienced >20% increases in OHCA (Fig. 1b). Irrespective of any potential local problem with available testing during that critical early month, nearly 90% (*n* = 30) of these cities were already in the highest attack rate category for documented COVID-19 cases/100,000 using the most recent CDC criteria. Moreover, 13 of these 34 cities had >1·5-fold increases in OHCA and three widely-recognized COVID-19 epicenters more than doubled their usual OHCA numbers (including a 2·5-fold increase in New York City). Meanwhile, many cities with relatively low COVID-19 prevalence in April such as Charleston, South Carolina (Figs. 1b, 2c) and Madison, Wisconsin (Figs. 1b, 2d), had no increases in OHCA compared to prior years or even lower frequencies (Fig. 1b).

Conversely, by June 2020 (Fig. 1d), cities with the highest rates of OHCA in April such as London, Milan, New York City, Detroit, St. Louis, and New Orleans (Figs. 1, 2, 3) returned to, or approached, historical levels after mitigating the local spread of COVID-19. In contrast, cities less impacted by COVID-19 and reporting their usual or lower numbers of OHCA in April, experienced marked increases during June 2020 as COVID-19 cases began to surge locally, particularly toward the end of that month and beginning of July. Meanwhile, cities like Albuquerque, Pittsburgh, Cleveland, Auckland, and Perth which were relatively less impacted by COVID-19 throughout April, May, and June, showed negligible percent changes in OHCA throughout the study period.

As noted previously, the UK, EU, Australian and New Zealand cities mirrored the U.S. experience including Milan and London showing marked increases, particularly in March and April 2020, but returning toward typical numbers following local control of cases while Perth and Auckland reported no increases during this period, again reflecting the small number of COVID-19 cases throughout all months studied in this report (Fig. 3).

## 4 Discussion

The findings in this populations-based observational study provide us with three main observations. First, SARS-CoV-2 infection appears to be strongly associated with OHCA, either as a possible primary presenting manifestation in some cases, or, more likely, as an early associated pathophysiological complication of the infection that can even occur among those who initially presented with less severe symptoms and were being managed outside of healthcare facilities [7,8,10,13]. It was first speculated that these cases may have been related to persons with typical acute coronary artery syndromes who were reticent to seek medical attention and later deteriorated into OHCA [7,8,10]. However, though a finite number of cases could be attributable to such circumstances, significant increases in OHCA were already being observed (and now definitively reported) in many large municipalities prior to most shelter-at-home directives being instituted, and even prior to more widespread public health warnings about the contagiousness, prevalence, and severity of the disease. Lock-down directives did not go into effect in New York City until the evening of March 22, yet that municipality already was experiencing many dozens of additional OHCA cases compared to prior years. New York City tallied a cumulative 500 OHCA case increase by month's end (only a week after lockdowns) [9]. In addition, to date, most of the individual cities’ studies have found that increases in OHCA were happening concurrently with increases in other EMS calls for presentations such as “flu-like” symptoms [7], [8], [9], [10]. Such reports indicated a mix of both milder and severe cases and some studies indicated that, retrospectively, there may have been some preceding fever or cough for a few prior days, but not in all cases [10]. Regardless, by most accounts, it did not appear that OHCA was the endpoint of a longstanding deterioration from a more severe and protracted disease process with multi-organ failure as seen in hospitalized COVID-19 patients who have been admitted and treated in intensive care units [15,23,24]. In addition, the cities that were not experiencing a high prevalence of COVID-19 in March and April 2020 such as Madison, St. Paul, Portland, Auckland, and Perth (Figs. 1a, 1b, 2d, 3) had very little change in the frequency of OHCA from prior years, particularly when compared to the majority of other large cities that were experiencing significant increases in OHCA and higher degrees of COVID-19 prevalence (Figs. 1a, 1b, 2, 3).

Moreover, in some cities such as Honolulu and Charleston, the frequency was even significantly lower than prior years despite the regional lockdowns during those months (Figs. 1a, 1b, 2c). The lower number of OHCA cases in these cities less impacted by COVID-19 (at the time) was likely attributable to diminished commuter populations and markedly depressed tourist volumes due to the lockdowns, quarantine policies and travel restrictions. This consideration may also further infer that, because of such restrictions, all of these large cities’ OHCA numbers might have been further escalated if their agencies were still responding to their usual pre-COVID populations and not the diminished populations secondary to the diminution of visitors, tourists and commuters [19,20].

Of particular note, after the highly-impacted cities such as London, Milan, Detroit, New Orleans, St. Louis, and New York City mitigated spread of the disease, the OHCA frequency fell accordingly (Figs. 1, 2a, 2b, 3). The methods-driven compositions of Fig. 2a and 2b were created to display the main results the study, namely the percent increases in OHCA cases being observed on a month-to-month basis in New York City and Detroit, not a day-to-day tally. As a result, the illustration creates the appearance that OHCA cases seemed to persist into late April as daily COVID-19 numbers were diminishing. However, as elegantly reported in other publications, the actual daily number of OHCA cases were dramatically escalating at the end of March into early April, and peaking well above the average for the month [[7], [8], [9],^25^,^26^]. Accordingly, daily numbers declined rapidly as the local COVID-19 prevalence dissipated in both cities [9,25].

Conversely, the cities (e.g., Madison, Charleston, Portland, Phoenix) experiencing only moderately elevated, unchanged or even lower frequencies of OHCA in April, then observed alarming increases in OHCA in late May or early June when COVID-19 was beginning to have large surges across those cities, particularly in the latter half of June and early July in Charleston, Madison and Phoenix (Figs. 1, 2c, 2d). In turn, cities less impacted by COVID-19 over the entire study period such as Pittsburgh, Cleveland, Perth, and Auckland experienced little operational impact in terms of OHCA compared to prior years (Figs. 1a-d, 3). While Perth and Auckland were singular sample cities representing their respective countries to balance the singular largest city representative cities from three high-impact European countries, their respective experiences also contributed an additional validation that mirrored the findings in the U.S. cities that had also experienced relatively low COVID-19 prevalence.

A second interesting observation in this study was that escalations in OHCA frequencies not only paralleled concurrent local COVID-19 prevalence, but that sudden surges in OHCA may also serve as an early foreshadowing or signal of pending COVID-19 surges in that community. Increases in OHCA were already beginning to evolve with resulting operational impact on the EMS systems in cities that were later identified as epicenters [[7], [8], [9],^26^]. The surge in OHCA cases were being observed locally at least a week or two before the large numbers of COVID-19 cases were being formally identified, both in the U.S. and Europe [9,26]. According to current World Health Organization statistics, fewer than 2 deaths were being attributed to COVID-19 in the U.S. by March 3 and less than 5000 as of April 1, 2020. However, beyond the 500-plus additional OHCA cases observed in New York City during March, the 50 cities collectively were already experiencing marked increases in OHCA that resulted in an exceptionally large number of additional associated deaths before March 31, 2020 (Fig. 1a) and those numbers accelerated during the first week of April [9].

Other investigators have also indicated that OHCA is directly related to COVID-19 [[7], [8], [9],^25^,^26^]. By inference, OHCA might be considered one of the myriad of chimeric presenting symptoms of COVID-19 or, more likely, one early-appearing complication of infection. Surges in OHCA cases would logically precede the typical time period when other persons with the more typically-described presentations would have sought medical care and received testing. Especially during the earlier phases of the study period, confirmation of infection was often delayed for days and, in turn, there were lags in cases being reported to public health officials and publicly announced. Therefore, beyond solely relying upon widespread testing and accumulation of individual patient data to identify evolving clusters, a surge and or cluster of OHCA cases might foreshadow a localized outbreak of COVID-19. This reinforces the concept that EMS incident data analyses can serve as another useful surveillance tool for the real-time monitoring of this public health crisis as well as for identifying and targeting COVID-19 cluster areas [27].

A third important implication of this study is the observation that these increases in OHCA and the resulting deaths were not counted among COVID-related deaths. As now reported in much greater detail by many of the individual participating cities, not only were there significantly increased numbers of OHCA during the pandemic outbreak, but also larger percentages of OHCA patients were found to be unsalvageable despite intensive resuscitation efforts [[7], [8], [9], [10],^25^,^26^,^28^]. The majority of these additional cases were pronounced dead, either on-scene after aggressive resuscitation efforts, or, in some cases, soon after emergency department arrival [[7], [8], [9], [10],^25^,^26^,^28^]. This pattern is consistent even within cities only mildly affected by COVID-19 [28]. Those same studies also provided further subgroup analyses emphasizing relative risks according to age, demographics, presenting cardiac rhythm, response intervals and treatment provided [[7], [8], [9], [10],^25^,^26^,^28^].

Of relevant note, very few of these OHCA patients were ever tested for COVID-19, even those later seen by medical examiners. Only two (4%) of the municipalities were attempting to test OHCA patients for COVID-19 and yet testing was still performed in less than half of those cities’ cases [17]. Overall, testing was rarely performed nationwide and particularly among the cities most affected by COVID-19 [9,25]. As noted previously, many of these additional OHCA cases and the resulting additional deaths were already beginning to add up before COVID-related deaths were being officially tallied in the U.S. As observed in this study, the 50 U.S. cities studied experienced over 4300 additional OHCA cases during April alone, nearly 2700 of which occurred in New York City where, similar to 96% of all U.S cities reporting, COVID-19 testing of these patients was rarely performed. By inference, there were many more of these untallied cases across other regions of the U.S. and numerous countries globally. Therefore, the total numbers of deaths attributable to COVID-19 are likely to be much higher than currently being reported.

With the compelling indications that the increases in OHCA cases may be directly related to COVID-19, it raises further questions regarding the pathophysiological mechanisms. Autopsy studies of in-hospital patients suggest most mortality emanates from diffuse alveolar damage accompanied by microvasculature thrombosis, microangiopathy and severe endothelial injury, all associated with a hypercoagulable state [[11], [12], [13], [14], [15],[29], [30], [31], [32], [33]]. Acute right ventricular dilatation is common but likely due to the well-described pulmonary disease in the most critically ill [34,35]. Although viral presence is seen within myocytes, there is a notable absence of widespread myocyte necrosis or interstitial lymphocytic infiltrate consistent with traditional myocarditis [30]. Nevertheless, for the OHCA cases, the suspicion remains that, in the progressive phases of early disease, the diffuse endotheliopathy, hypercoagulable state, and associated microcirculation thrombosis likely result in diffuse myocardial dysfunction and ensuing OHCA [15,31,32]. While COVID-19 systemic inflammation and cytokine storm can lead to coronary plaque instability, rupture and resulting type-I ST-segment elevation, myocardial infarction (STEMI) and malignant dysrhythmias, such systemic inflammation, is usually found in latter stages of COVID-19 among very sick persons already hospitalized for some period of time and not the current cohort being evaluated here [10].

COVID-19 patients do have increased risk for malignant dysrhythmias due to sympathetic stimulation and marked proinflammatory effects that enhance development of long QTc and Torsades de Pointes, often compounded by certain medications [10,34]. However, data from New York City, Milan, London, Paris, and other populations demonstrated disproportionate numbers of OHCA patients presenting with non-shockable, brady-asystolic electrocardiographic tracings, again suggesting hypoxic demise or circulatory compromise (pump failure, massive PTE) [[7], [8], [9], [10], [11], [12], [13], [14], [15],^25^,^26^,^28^,^33^,^35^,^36^]. Therefore, the mechanism is less likely to be a classic ventricular dysrhythmia, but rather one of myocardial dysfunction and ensuing OHCA emanating from a number of overlapping mechanisms likely related to a widespread endotheliopathy, hypercoagulable states, and micro and macro-thrombosis. Although much of the COVID-19 PTE literature was derived from critically-ill hospitalized patients, others have now documented the presence of PTE, including massive PTE and sudden cardiac death, in non-critically-ill patients, and even outpatients [13,14].

Regardless of etiology, increased frequencies of COVID-19 cardiac arrests have placed significant operational and resource burdens on EMS systems [2], [3], [4], [5], [6]. The sheer number of responding apparatus and personnel directly involved in a given resuscitation attempt precludes availability of numerous responders for other 9–1–1 (or equivalent) calls. More importantly, OHCA incurs the stressors of seamlessly performing a highly-choreographed bundle of care resembling a pit crew approach conducted in a volatile, extremely high-pressure situation surrounded by highly-distraught family members and other bystanders, now compounded by a marked risk for exposure to a deadly disease and use of high-level personal protective equipment and procedures that may impede patient care efficiencies during these time-dependent crises [4,5,26].

Beyond response intervals and time taken to locate a given patient at a home, high-rise or workplace, OHCA involves a minimum of 30 to 40 min on-scene and significant additional time to evacuate and transport the patient [2,3]. Even if efforts are terminated on-scene, OHCA cases require highly-detailed medical record documentation, re-stocking of equipment and numerous medications and, most importantly, sensitive interactions with family members. Therefore, operationally, OHCA incidents consume unusual amounts of resources and typically keep ambulances and personnel away from other front-line emergency care responses for as long as two hours or more in many cases. While constituting only 1% of all EMS responses (pre-COVID), OHCA can ordinarily impact 15–20% of metropolitan EMS system resources on any given day, particularly in terms of time consumed away from other EMS responses [1], [2], [3]. In essence, COVID-related increases in OHCA have expended an extremely disproportionate amount of time, work efforts, emotional stress, and have further augmented physical and professional risks.

Compounding the operational strain, most responding EMS personnel maintain emotional concerns in terms of being exposed to the contagion particularly with airway management and positive pressure ventilation techniques required in OHCA resuscitation efforts [26,37]. COVID-19 amplified these stressors, including exceptional attention to PPE donning and doffing, especially in the early days when more definitive information about the SARS-CoV-2 virus was so sparse.

The greatest limitation to this study is that COVID-19 infection was not formally documented in these OHCA cases. The suggestion that the associated increases in OHCA incidents were directly caused by COVID-19 cannot be confirmed definitively within the current methodology. However, the observations that the frequency of OHCA immediately heralded and paralleled the concurrent level of detected SARS-CoV-2 prevalence in the given metropolitan area is very compelling, particularly as the frequency rose and fell accordingly in many cities [8,9,25,26]. This proposition is most strongly supported by early publications about OHCA in Lombardia, Italy, which demonstrated the highest increases in OHCA within the districts of that region with the highest number of COVID-19 cases (Lodi and Cremona) while those with fewer COVID-19 cases (Pavia and Mantua) did not see such increases [7,10].

Additionally, the current study did not attempt to address details such as the presenting cardiac rhythm, demographics, outcome or forensic information. Those data have been reported elsewhere or are part of upcoming publications [[7], [8], [9],^25^,^26^,^28^,^36^]. The main targeted aim of this current study was to quantitate and document, as a hypothesis-generating observation, the presumptive inferential relationships between local COVID-19 prevalence and the frequency of OHCA.

One final limitation here was that the actual prevalence of COVID-19 in a community was likely a best estimate. Beyond asymptomatic carriers and milder, unreported cases, the COVID-19 numbers here only entailed confirmed cases. Also, reported test confirmations, hospitalizations, and deaths lagged well-beyond the time of exposure and disease onset. Early on in the pandemic and during much of this investigation's study period, testing was very limited and results were not reported rapidly. Therefore, parallel elevations and falls in OHCA numbers and reported COVID-19 cases do not constitute an exact science. However, the identified parallel relationship between COVID-19 and frequency of OHCA still remains a very reasonable conclusion that can be readily based on other common and well-reported epidemiological observations.

## Data sharing statement

Aggregate, de-identified raw data for each cities’ population-based, out-of-hospital cardiac arrest statistics for 2018, 2019, and 2020 are available in standard spreadsheets upon request. Editors may request these data from the primary authors through the corresponding author.

## Author contributions

• Kevin E. McVaney, MD: Conceptualisation, methodology, supervision, writing and reviewing and project administration (supervisor and overseer of IRB submitter)
• Paul E. Pepe, MD, MPH: Corresponding author, conceptualisation, supervision, data validation, writing (original draft and reviewing and editing)
• Lauren M. Maloney, MD: Literature search, primary assimilator of data collection and curation, software and data validation, figure development, writing and editing of final manuscript
• E. Stein Bronsky, MD: Project administration, resources, data collection and assimilation, conceptualisation, methodology review, data validation, reviewing and editing the manuscript
• Remle P. Crowe, PhD: Data validation, methodology, supervision of formal statistical analysis, data curation and editing and reviewing the final manuscript.
• James J. Augustine, MD: Project administration, gathering relevant population information and recruitment of investigators and their data as well as conceptualisation of the project, guidance in reviewing and editing the manuscript
• Sheaffer O. Gilliam, MD: Conceptualisation, literature review, project administration (submitter of institutional review board documents).
• Glenn H. Asaeda, MD: Conceptualisation, methodology, major data contributor
• Marc Eckstein, MD, MPH: Conceptualisation, methodology, major data contributor, editing of abstract
• Amal Mattu, MD: Literature review, methodology, writing, editing and reviewing final manuscript
• Roberto Fumagalli, MD: Conceptualisation, methodology, major data contributor, editing
• Tom P. Aufderheide, MD: Supervision, review of methodology, editing and final manuscript review and recommendations
• Michael T. Osterholm, PhD, MPH: Supervision, senior author, review and methodological context and significance of findings
• Drs. Maloney, Crowe and Pepe cross-checked and verified the data received from the various sites.
• All authors had full access to all of the raw data in the study and accept responsibility for its integrity and all have reviewed the contents of the paper.
• No external medical editor or writer was involved. The work product is solely the effort of the authors.

## Funding

No funding was involved. Participating cities directly provided de-identified aggregate data collected routinely for standard quality assurance functions.

## Declaration of Competing Interest

None of the authors or contributing investigators have any conflict of interest with respect to this observational, epidemiological, population-based cross-sectional research study which was based on routinely-collected public agency data sets.

## Appendix Supplementary materials

Image, application 1

## References

[1] Merchant R.M., Topjian A.A., Panchal A.R. (2020). Part 1: executive summary: 2020 American Heart Association guidelines for cardiopulmonary resuscitation and emergency cardiovascular care. Circulation.

[2] Chan H.K., Okubo M., Callaway C.W., Mann N.C., Wang H.E. (2020). Characteristics of adult out-of-hospital cardiac arrest in the National Emergency Medical Services Information System. JACEP Open.

[3] Pepe P.E., Aufderheide T.P., Lamhaut L. (2020). Rationale and strategies for development of an optimal bundle of management for cardiac arrest. Crit Care Explor.

[4] Banerjee P.R., Ganti L., Pepe P.E., Singh A., Roka A., Vittone R.A. (2019). Early on-scene management of pediatric out-of-hospital cardiac arrest can result in improved likelihood for neurologically-intact survival. Resuscitation.

[5] Lerner E.B., Newgard C.D., Mann N.C. (2020). Effect of the coronavirus disease 2019 (COVID-19) pandemic on the U.S. emergency medical services system: a preliminary report. Acad Emerg Med.

[6] Jaffe E., Sonkin R., Strugo R., Zerath E. (2020). Evolution of emergency medical calls during a pandemic - An emergency medical service during the COVID-19 outbreak. Am J Emerg Med.

[7] Baldi E., Sechi G.M., Mare C. (2020). Out-of-hospital cardiac arrest during the Covid-19 outbreak in Italy. New Engl J Med.

[8] Marijon E., Karam N., Jost D. (2020). Out-of-hospital cardiac arrest during the COVID-19 pandemic in Paris, France: a population-based, observational study. Lancet Public Health.

[9] Lai P.H., Lancet E.A., Weiden M.D. (2020). Characteristics associated with out-of-hospital cardiac arrests and resuscitations during the novel coronavirus disease 2019 pandemic in New York City. JAMA Cardiol.

[10] Baldi E., Sechi G.M., Mare C. (2020). COVID-19 kills at home: the close relationship between the epidemic and the increase of out-of-hospital cardiac arrests. Eur Heart J.

[11] Fraser D.D., Patterson E.K., Slessarev M. (2020). Endothelial injury and glycocalyx degradation in critically ill coronavirus disease 2019 patients: implications for microvascular platelet aggregation. Crit Care Explor.

[12] Wichmann D. (2020). Autopsy Findings and venous thromboembolism in patients with COVID-19. Ann Intern Med.

[13] Gervaise A., Bouzad C., Peroux E., Helissey C. (2020). Acute pulmonary embolism in non-hospitalized COVID-19 patients referred to CTPA by emergency department. Eur Radiol.

[14] Mestre-Gómez B., Lorente-Ramos R.M., Rogado J. (2020). Incidence of pulmonary embolism in non-critically ill COVID-19 patients. predicting factors for a challenging diagnosis. J Thromb Thrombolysis.

[15] Hranjec T., Estreicher M., Rogers B. (2020). Integral use of thromboelastography with platelet mapping to guide appropriate treatment, avoid complications and improve survival of patients with COVID-19-related coagulopathy. Crit Care Expl.

[16] Metropolitan EMS Medical Directors Global Alliance (2020). History and current status of the “Eagles” conferences.

[17] Gates H. (2020). Resident Eagle: inside the war room – medical directors take on COVID-19. EMS World.

[18] Pepe P.E., Copass M.K., Fowler R.L., Cone DC, Fowler R, O'Connor RE (2009). Medical direction of emergency medical services systems. Emergency Medical Services: Clinical Practice and Systems Oversight.

[19] McPherson T.N., Brown M.J. (2003). Estimating daytime and nighttime population distributions in U.S. cities for emergency response activities. https://ams.confex.com/ams/pdfpapers/105209.pdf.

[20] McKenzie B., Koerber W., Fields A., Benetsky M., Rapino M. (2020). Commuter-adjusted population estimates: acs 2006-10. https://www.census.gov/topics/employment/commuting/guidance/calculations.html.

[21] Centers for Disease Control and Prevention COVID Data Tracker https://www.cdc.gov/coronavirus/2019-ncov/travelers/how-level-is-determined.html. Last accessed 13 February 2021.

[22] Coronavirus in the U.S.: Latest map and case count. https://www.nytimes.com/interactive/2020/us/coronavirus-us-cases.html Last accessed 13 February 2021.

[23] Shi S., Qin M., Shen B. (2020). Association of cardiac injury with mortality in hospitalized patients with COVID-19 in Wuhan, China. JAMA Cardiol.

[24] Xu S., Ma X., Xu Z. (2020). In-hospital cardiac arrest outcomes among patients with COVID-19 pneumonia in Wuhan, China. Resuscitation.

[25] Nickles A.V., Oostema A., Allen J. (2021). Comparison of out-of-hospital cardiac arrests and fatalities in the metro Detroit area during the COVID-19 pandemic with previous-year events. JAMA Netw Open.

[26] Fothergill R.T., Smith A.L., Wrigley F., Perkins G.D. (2021). Out-of-hospital cardiac arrest in London during the COVID-19 pandemic. Resuscitation Plus.

[27] Prahlow S.P., Atrubin D., Culpepper A., Hamilton J.J., Sturms J., Card K. (2019). Approach to onboarding emergency medical services (EMS) data into a syndromic surveillance system. Online J Public Health Inform.

[28] Uy-Evanado A., Chugh H.D., Sargsyan A. (2021). Out-of-hospital cardiac arrest response and outcomes during the COVID-19 pandemic. J Am Coll Cardiol Clin Electrophysiol.

[29] Fox S.E., Akmatbekov A., Harbert J.L., Li G., Quincy Brown J., Vander Heide R.S (2020). Pulmonary and cardiac pathology in African American patients with COVID-19: an autopsy series from New Orleans. Lancet Respir Med.

[30] Lindner D., Fitzek A., Bräuninger H. (2020). Association of cardiac infection with SARS-CoV-2 in confirmed COVID-19 autopsy cases. JAMA Cardiol.

[31] Schaller T., Hirschbühl K., Burkhardt K. (2020). Postmortem examination of patients with COVID-19. JAMA.

[32] Ackermann M., Verleden S.E., Kuehnel M. (2020). Pulmonary vascular endothelialitis, thrombosis, and angiogenesis in Covid-19. New Engl J Med.

[33] Madjid M., Safavi-Naeini P., Solomon S.D., Vardeny O. (2020). Potential effects of coronaviruses on the cardiovascular system: a review. JAMA Cardiol.

[34] Lazzerini P.E., Boutjdir M., Capecchi P.L. (2020). COVID-19, Arrhythmic Risk, and Inflammation: mind the Gap!. Circulation.

[35] Ali S., Mathew S., Pappachan J.M. (2020). Acute cor pulmonale from saddle pulmonary embolism in a patient with previous COVID-19: should we prolong prophylactic anticoagulation?. Int J Infect Dis.

[36] Chan P.S., Girotra S., Tang Y., Al-Araji R., Nallamothu B.K., McNally B. (2020). Outcomes for out-of-hospital cardiac arrest in the United States during the coronavirus disease 2019 pandemic. JAMA Cardiol.

[37] Sayre M.R., Barnard L.M., Counts C.R., Drucker C.J., Kudenchuk P.J., Rea T.J., Eisenberg M.S. (2020). Prevalence of COVID-19 in out-of-hospital cardiac arrest: implications for bystander cardiopulmonary resuscitation. Circulation.

