# Supplementary material for: The relationship of large city out-of-hospital cardiac arrests and the prevalence of COVID-19
Source: eClinicalMedicine. 2021 Apr 7;34:100815. doi: 10.1016/j.eclinm.2021.100815 (PMC8102707; doi:10.1016/j.eclinm.2021.100815)
Supplement: Supplementary file 1 [file mmc1.pdf]

APPENDIX – Collaborating Study Group Contributing Investigators

The Contributing Investigators from the *Metropolitan EMS Medical Directors Global Alliance* ("Eagles") and their affiliated collaborators have provided de facto consent to publish their information by submission of their agency's data and by participating in a formal survey and request for these applicable data. They also participated in internal review of the primary findings.

The contributing study group investigators included the following persons:

- *American College of Emergency Physicians*: Sandra M. Schneider, MD (forum co-host)
- *National Association of EMS Physicians*: Michael K. Levy, MD; Jose Cabanas, MD (President and President-Elect and forum co-hosts) -- also see Anchorage & Raleigh below.
- *National Association of Emergency Medical Technicians*: Craig A. Manifold, DO (forum co-host; now deceased)
- Greater Broward EMS Medical Directors Association: Peter M. Antevy, MD; Randy S. Katz, DO & James P. Roach, DO (satellite conference hosts)

Albuquerque, NM: Kimberly Pruett, MD
Anchorage, AK: Michael Levy MD
Atlanta, GA: Lekshmi Kumar, MD
Auckland, NZ: Craig Ellis, MD
Austin-Travis County, TX: Mark Escott, MD, MPH and Jeff Hayes, LP, MPH
Baltimore, MD: Benjamin Lawner, DO
Berlin, Germany: Stefan Poloczek, MD
Boston, MA: K. Sophia Dyer, MD
Capetown, South Africa: Shaheem de Vries, MD
Charleston, SC: David M. French, MD
Charlotte, NC: Doug Swanson, MD
Chicago, IL: Joseph Weber, MD; Katie Tataris, MD and Eddie Markul, MD
Cleveland, OH: Thomas Collins, MD
Colorado Springs, CO: E. Stein Bronsky, MD and Mark Warth, NRP
Columbus, OH: Robert A. Lowe, MD
Dallas, TX: S. Marshal Isaacs, MD and Ray Fowler, MD
Dayton, OH: David N. Gerstner, EMT-P
Denver, CO: Kevin McVaney, MD
Detroit, MI: Robert B. Dunne, MD and Damon Gorelick, EMT-P
Fort Lauderdale, FL: James P. Roach, DO
Fort Worth, TX: Veer D. Vithalani, MD
Honolulu, HI: Elizabeth A. Char, MD and Sean Covant, MD
Houston, TX: David E. Persse, MD
Indianapolis, IN: Daniel O'Donnell, MD
Jacksonville, FL: Brad Elias, MD
Kansas City, MO: Erica Carney, MD
Las Vegas, NV: David Slattery, MD
Little Rock, AR: Chuck Mason, MD
London, UK: Fenella Wrigley, FRCEM and Rachael T. Fothergill, BSc (Hons), PhD
Los Angeles, CA: Marc K. Eckstein, MD
Madison, WI: Megan Gussick, MD
Memphis, TN: Joe Holley, MD

Melbourne, Australia: Stephen Bernard, MD, David Anderson, MD, MStJ, MBChB
Miami, FL: Paul Adams, DO and Armando Clift, MD
Milan, Italy: Roberto Fumagalli, MD
Milwaukee, WI: Benjamin W Weston, MD, MPH
Minneapolis, MN: Marc Conterato, MD and Nicholas Simpson, MD
Nashville, TN: Corey Slovis, MD
New Orleans, LA: Megan Marino, MD and Emily M Nichols, MD
New York City, NY: Glenn Asaeda, MD
Oklahoma City, OK: Jeffrey M. Goodloe, MD
Orlando, FL: Christian C. Zuver, MD; Alexa Rodriguez, MD and Amy Souers, MD
Paris, France: Pierre A. Carli, MD
Perth, Australia: Paul Bailey, MB, FACEM, PhE
Philadelphia, PA: C. Crawford Mechem, MD
Phoenix, AZ: Garth Gemar, MD
Pittsburgh, PA: Ronald Roth, MD
Portland, OR: Jonathan Jui MD, MPH
Raleigh, NC: Jose G. Cabanas, MD, MPH
Regional Louisiana/Texas / Acadian Ambulance: Chuck Burnell, MD
Sacramento, CA: Kevin E. Mackey MD
Salt Lake City, UT: Scott T Youngquist, MD, MS
San Antonio, TX: David A. Miramontes, MD and C.J. Winckler, MD
San Diego, CA: Christopher Kahn, MD, MPH and J. Joelle Donofrio-Odmann DO
San Francisco, CA: Christopher B. Colwell, MD
San Jose, CA: Marc Gautreau, MD and Eli Carrillo, MD
Seattle, WA: Michael Sayre, MD and Andrew McCoy, MD
South East Coast Ambulance Service NHS Foundation Trust,
West Sussex, UK: Fionna P. Moore, MD, MBE
Singapore, SG: Marcos Ong En Hock, MD
St. Louis, MO: William S. Gilmore, MD
St. Paul, MN: Ralph Frascone, MD
Tampa, FL: Angus Jameson, MD; Rachel Semmons, MD; Andrew Thomas, MD; Michael Lozano, MD
Tucson, AZ: Joshua Gaither, MD
Tulsa, OK: Jeffrey M. Goodloe, MD
Washington, DC: Robert P. Holman, MD
West Palm Beach, FL: Kenneth A Scheppke, MD
Wichita, KS: John Gallagher, MD
